# Explicating the responses of NDVI and GDP to the poverty alleviation policy in poverty areas of China in the 21^st^ century

**DOI:** 10.1371/journal.pone.0271983

**Published:** 2022-08-15

**Authors:** ZeMeng Fan, XuYang Bai, Na Zhao

**Affiliations:** 1 State Key Laboratory of Resources and Environment Information System, Institute of Geographical Sciences and Natural Resources Research, Chinese Academy of Sciences, Beijing, 100101, China; 2 College of Resources and Environment, University of Chinese Academy of Sciences, Beijing, 100049, China; 3 Jiangsu Center for Collaborative Innovation in Geographical Information Resource Development and Application, Nanjing, 210023, China; Sichuan Agricultural University, CHINA

## Abstract

The economy in the poverty-stricken areas of China has grown rapidly in response to poverty alleviation policies in the 21st century. To explicate the response of the eco-environment to rapid economic growth in the 14 contiguous areas of dire poverty in China, we developed a method of evaluating the impact of poverty alleviation policies on ecological health. Based on the yearly data of gross domestic product (GDP) per capita and normalized difference vegetation index (NDVI) from 2000 to 2019, the dynamic changes in NDVI and GDP were calculated, and the development patterns in the 14 contiguous areas of dire poverty were evaluated and classified. The results show that both annual GDP per capita and average annual NDVI exhibited an increasing trend, increasing by 43.81% and 0.84% per year, respectively. The development of the 14 contiguous areas of dire poverty all presented a coordinated and sustainable (A) development pattern during the period from 2000 to 2019. The consistency of economic and ecological health development between 2000 and 2013 was less than that between 2014 and 2019. Moreover, the result indicates that it is necessary to make timely adjustments to poverty alleviation strategies based on the positive consistency between economic growth and ecological health.

## Introduction

Eliminating poverty has long been the common goal of humankind [1–4]. From ancient times to the present, the development of human society has been limited to poverty alleviation and the series of problems derived from hunger, disease, and harsh environments [5,6]. With the rapid development of society, science and technology, the share of the world’s population living in extreme poverty declined from 15.70% to 10.00% between 2000 and 2015, but the pace of global poverty reduction has been decelerating. Thus, poverty is still a very important and difficult problem that all countries in the world, especially developing countries, urgently need to solve [7–10]. Various countries and regions have adopted a variety of poverty reduction measures in accordance with the corresponding conditions in poverty-stricken areas [11–14]. However, with the implementation of a series of poverty alleviation policies in separate countries or regions, different changes in ecological health and sustainability have taken place in distinct poverty areas [15,16], and rapid economic growth has led to ecological degradation [17] in developing countries or poverty areas.

Economic growth is a measurement of poverty alleviation commonly used to evaluate the effect of poverty alleviation measures in most economic research fields [18,19]. Economic growth is beneficial to closing the poverty gap and eliminating poverty; however, the complicated relationship between economic growth and ecological health should not be ignored [15,20–22]. Related studies indicate that the complexity of economic-ecological environmental dynamics is damaging ecological health and sustainability in many areas of the world [23]. Some regions are eager to eliminate poverty and put economic development in the forefront, resulting in regional economic development often at the expense of the environment, that is, pollution first and then governance. Such an economic growth pattern threatens ecological health and sustainability directly [16]. Ecosystem degradation caused by economic growth or economic compensation for ecological protection and restoration is a precise consumption of sustainable development. For example, the impact of agricultural development on the ecological environment shows that the increase in agricultural income exacerbated ecological degradation in the 11 countries of Central and West Africa between 1996 and 2015 [24]. Research on the relationship between economic growth and CO_2_ emissions based on Vietnam’s annual reported data shows that economic growth and CO_2_ emission increases showed a significant positive correlation in Vietnam between 1974 and 2016 [17]. In South Africa, rapid economic growth and urbanization have directly led to an increase in energy demand, which has had a significant positive correlation with eco-environmental system degradation since the 1990s [24].

In addition, many efforts have been made to speed up economic development in poverty-stricken areas without damaging ecological health or sustainability, which means that the balance between economic growth and ecological protection has been fully considered in the process of implementing poverty alleviation policies [3,4]. For example, a good balance between economic growth and biodiversity conservation has been maintained by rapidly expanding nature reserves in the process of poverty alleviation in Madagascar [25]. In Huambo, Ethiopia, the introduction of a suitable clean technology has not only been beneficial for alleviating poverty but has also helped restore the degraded ecological environment [26]. Ecological projects in the karst area of Southwest China have also made good progress toward maintaining the balance between rapid economic growth and ecological restoration since the beginning of the 21^st^ century [27,28].

Poverty research is critical, particularly in assessing the effectiveness of poverty-reduction policies. Therefore, it is important to employ rigorous evaluation methods to better understand the effects of poverty alleviation policies in various contexts. At present, three methods are commonly used, i.e., descriptive assessment methods, quantitative assessment methods and mixed evaluation methods. The general practice of the descriptive evaluation method is to provide representative descriptive results by using random sampling survey data and estimate the impact of policy intervention [29–33]. Zou et al. [34] used three data accumulation methods (field investigation, key information interview, face-to-face and questionnaire survey) to select representative data to evaluate the effect of the relocation poverty alleviation policy in Tongyu County, Jilin Province, China. The common method of quantitative evaluation uses a series of mathematical functions (such as regression) to approximate the causal relationship between poverty alleviation policies and the affected factors and draws a conclusion about whether the policy effect is rigorous or not [35,36]. Han and Gao [37] used regression discontinuity design (a quasi-experimental method) to approximate the causal relationship between Korean national policies and household consumption patterns and drew strict conclusions on policy effects. The mixed evaluation method is generally a combination of qualitative and quantitative methods. The impact of policy intervention can be quantified by selecting factors and assigning weights to them [38,39]. Singh and Chudasama [40] identified the key factors in reducing poverty in India using a fuzzy cognitive map (FCM), which is used to demonstrate causal reasoning. Then, they evaluated the effectiveness of existing poverty alleviation methods using FCM simulation.

China, a developing country with the greatest number of poor people in the world, has implemented a series of policies designed to eliminate poverty, which has led to a reduction in the number of poor people from 689 million to 100 million between 1990 and 2013 [15]. Since 2013, a targeted poverty alleviation policy has been implemented, with 1.6 trillion in funds invested by the Chinese government to eliminate poverty. The last 100 million poor people in China were lifted out of poverty in 2020, which made a significant contribution to the goal of global poverty reduction. Against the background of China’s rapid economic growth, the changing trend of ecological health has become a hot scientific topic. Fourteen contiguous areas of dire poverty are typical poverty areas with poor living conditions [41], frequent natural disasters [42], weak economic foundations, and poor per capita net income of farmers and per capita regional GDP [43–46] in the world, in which a large number of poor people are usually concentrated.

We proposed a method of evaluating the effect of poverty alleviation policies on ecological health to better assess the impact of poverty alleviation policies on ecological health in fourteen contiguous areas of dire poverty. The goal was to investigate the impact of poverty alleviation policies on ecological health from the perspective of economic growth. This paper intends to use this method to evaluate the impact of poverty alleviation policies on ecological health and the synergy of economic and ecological health development in fourteen contiguous areas of dire poverty from 2000 to 2019 using gross domestic product (GDP) per capita and normalized difference vegetation index (NDVI) data. The research results are expected to determine whether China’s poverty alleviation policy is sustainable and to provide useful references for other regions.

## Materials and methods

### Study context

**Fig 1:**
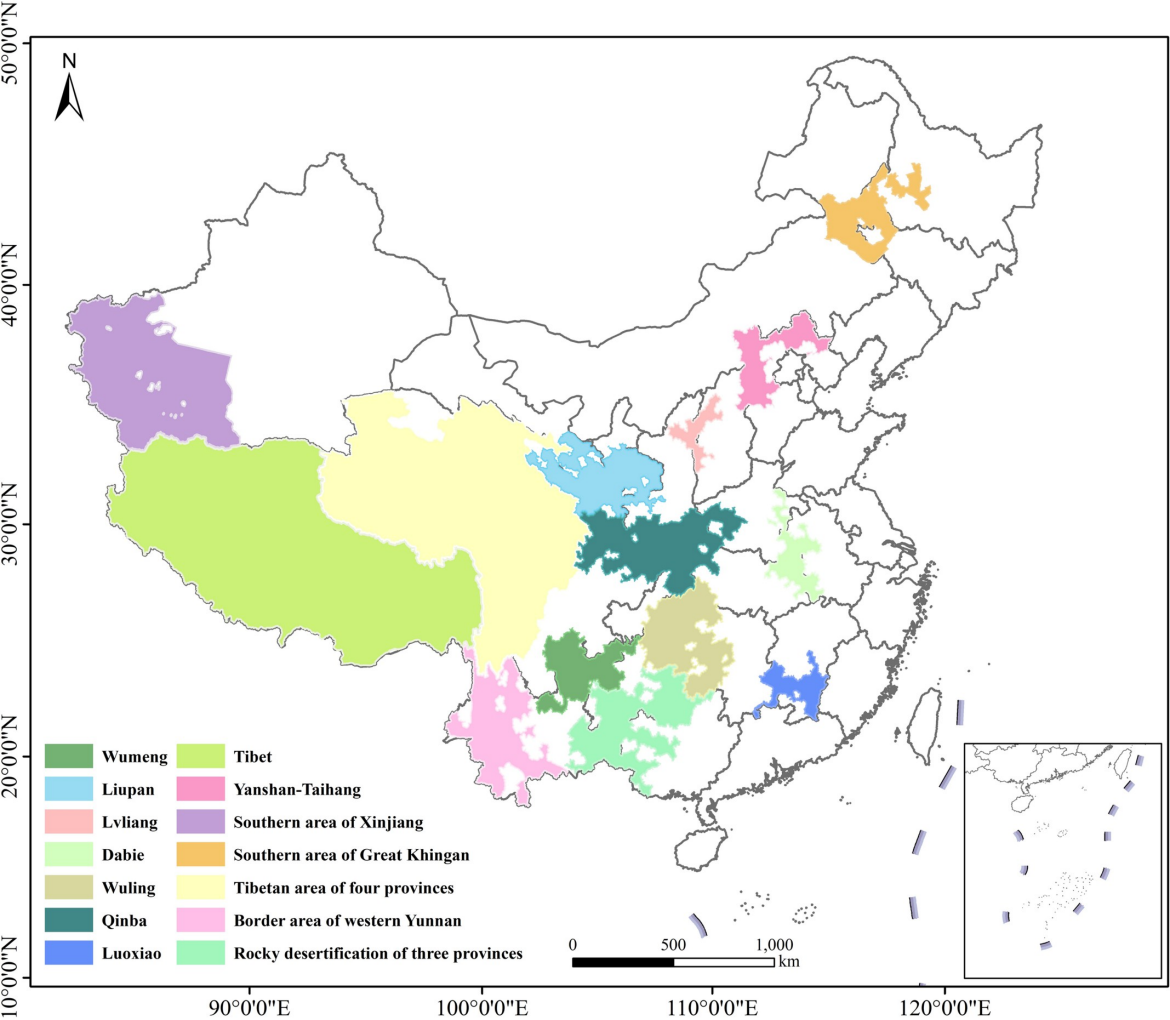
The spatial distribution of 14 contiguous poverty areas in China.

The targets of China’s poverty alleviation policy have ranged from poverty targeting and poverty alleviation under the reform of the rural economic system (1978–1984) to impoverished counties (1985–2000), impoverished villages (2000–2010), and finally, regional peace village-to-household targeting (2011–2020) [47–49]. During the period of regional targeting and village-to-household targeting, China focused on fourteen contiguous areas of dire poverty with special difficulties as the main battlefield of poverty alleviation. Based on the *outline for poverty alleviation and development in rural China (2011–2020)* and the principle of "centralization and connection, highlighting key points, overall national planning and complete zoning", the poverty-stricken areas of China were classified into fourteen contiguous areas of dire poverty (Fig 1) that included Wumeng, Liupan, Southern area of Xinjiang (Kashgar, Hotan and Kizilsu Kirgiz Autonomous Prefecture), Lvliang, Tibetan area of four provinces (Qinghai-Tibetan, Sichuan-Tibetan, Yunnan-Tibetan and Gansu-Tibetan area), Southern area of Great Khingan, Dabie, Wuling, Border area of western Yunnan, Rocky desertification of three provinces (Yunnan, Guangxi and Guizhou), Yanshan-Taihang, Qinba Mountain, Luoxiao, and Tibet, which involve 680 counties of China (http://www.cpad.gov.cn/).

### Data collection and processing

The research data in this study include the normalized difference vegetation index (NDVI) and gross domestic product (GDP) per capita. MODIS NDVI data come from the USGS, with a spatial resolution of 250 meters (m) and a temporal resolution of 16 days. The date range is from February 2000 to December 2019 (https://lpdaac.usgs.gov). The monthly and annual NDVI data of 14 continuous poverty areas from 2000 to 2019 were obtained by the maximum value composite [50] of MODIS NDVI data on the GEE platform. The GDP per capita data of 680 counties belonging to the 14 contiguous areas of dire poverty from 2000 to 2019 were collected from the provincial statistical yearbook of China (http://www.stats.gov.cn/tjsj/), and the annual GDP per capita in every contiguous poverty area was calculated.

### A method of evaluating the effect of poverty alleviation policies on ecological health

Achieving sustainable development through the balanced integration of the three pillars of economic development, social development, and environmental protection and conservation is very important in poverty alleviation. Unsustainable development threatens not only the ecological health but also the livelihoods of people. Therefore, it is critical to determine whether poverty alleviation policies are compatible with long-term development goals. In this paper, we propose a method for evaluating the effect of poverty alleviation policies on ecological health. The goal of this method is to investigate the impact of poverty alleviation on ecological health from the perspective of economic growth caused by poverty alleviation. GDP per capita is one of the important indicators to measure the level of economic development [51], and NDVI is also one of the most commonly used indicators to reflect vegetation status [42–56], so we chose these two indicators to represent economic and ecological health for analysis. We can judge the response of ecological health to rapid economic development in poor areas by analyzing the relationship between GDP per capita and NDVI and then evaluate the effect of poverty alleviation policies on ecological health.

**Fig 2:**
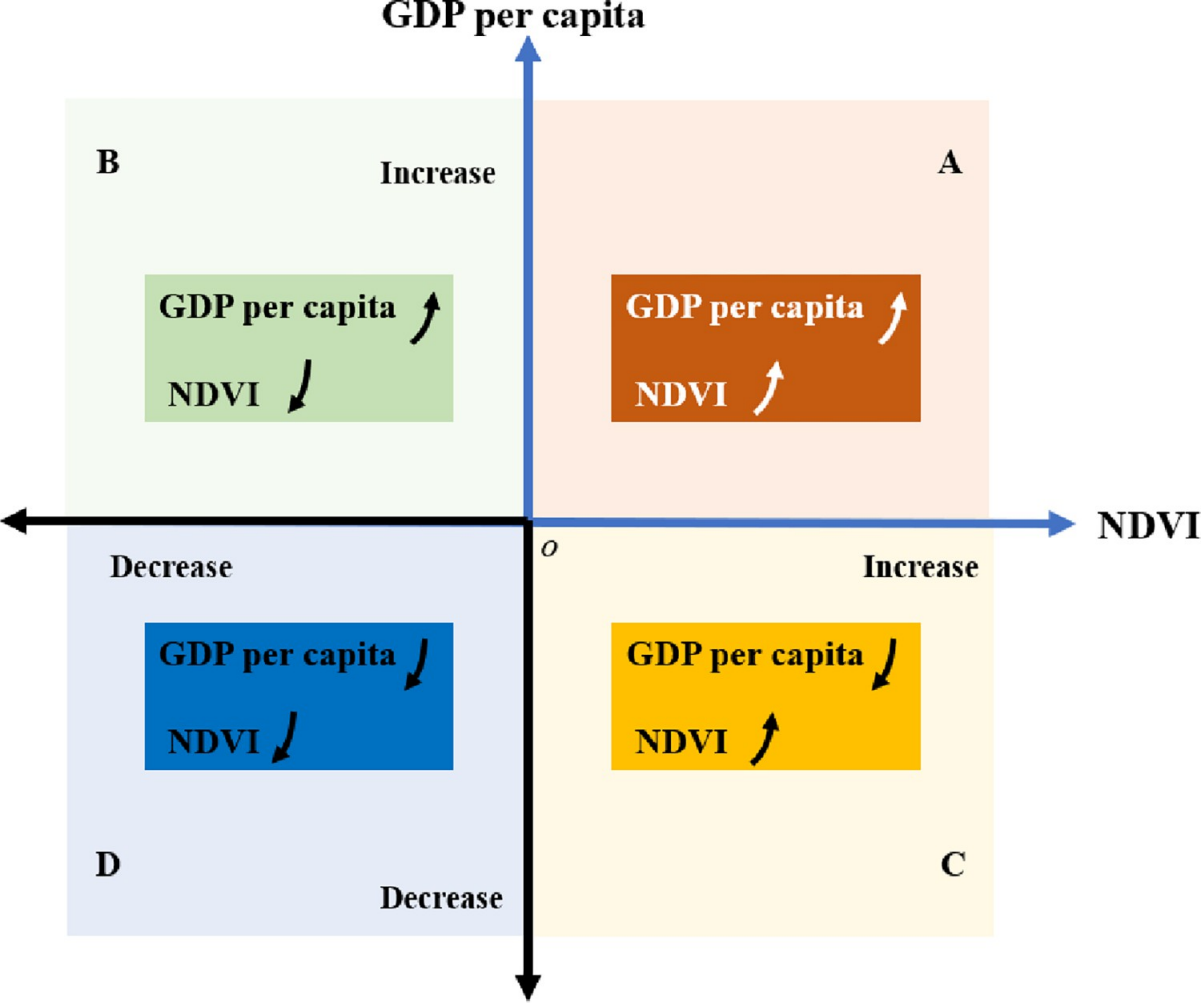
The framework of the evaluation of poverty alleviation policy on ecological health.

The change trends of GDP per capita and NDVI are divided into four directions of A, B, C, and D (Fig 2), which represent the four development patterns between the economy and the eco-environment: coordinated sustainability (A), economic-growth focused (B), eco-environmental-dominant development (C), and morbid development (D).

The coordinated and sustainable (A) development pattern denotes that the regional economy and environment have both been well developed under the implementation of poverty alleviation policies. In practical applications, the development patterns can be judged by the positive and negative development slopes of GDP per capita and NDVI (Table 1). It is classified as a coordinated sustainability (A) development pattern if the slopes of GDP per capita and NDVI are greater than zero. The economic growth (B) development pattern denotes that only economic development is focused on under the implementation of poverty alleviation policies, while the development of the environment is ignored. It is classified as economic-growth focused (B) development pattern if the slope of GDP per capita is greater than zero and the slope of NDVI is less than zero. Eco-environmental-dominant development (C) denotes that the poverty alleviation policies only focus on the environment, while the economy is ignored. It is classified as the eco-environmental-dominant development (C) pattern if the slope of GDP per capita is less than zero and the slope of NDVI is greater than zero. Morbid development (D) denotes that development of the regional economy and environment have both worsened under the implementation of poverty alleviation policies. It is classified as dominated by a morbid development (D) pattern if the slopes of GDP per capita and NDVI are less than zero. The Pearson correlation coefficient [57] is widely used to measure the correlation between two variables and can be used as an optimization criterion to judge the synchronism between two variables. To analyze the correlation between GDP growth and NDVI change, the Pearson correlation coefficient was used to calculate the correlation coefficient (*r*_*NDVI*,*GDP*_) between GDP per capita and NDVI, which can be formulated as follows:

$$r_{NDVI,GDP}=\frac{\sum_{i=1}^{n}\left(NDVI_{i}-\bar{NDVI}\right)(GDP_{i}-\bar{GDP})}{\sqrt{\sum_{i=1}^{n}\left(NDVI_{i}-\bar{NDVI}\right)}\sqrt{\sum_{i=1}^{n}\left(GD{P_{i}}_{i}-\bar{GDP}\right)}}$$ (1)

where *n* is the study period; *i* is the time, from 1 to *n*; *n* is the total number of years; *NDVI*_*i*_ is the NDVI value in year *i*; *GDP*_*i*_ is the GDP per capita in year *i*; $\bar{NDVI}$ is the average annual NDVI; and $\bar{GDP}$ is the average GDP per capita. The value of *r*_*NDVI*,*GDP*_ ranges from -1 to 1; if *r*_*NDVI*,*GDP*_ = 0, there is no correlation between the NDVI change and the GDP per capita increase; if *r*_*NDVI*,*GDP*_<0, it shows that the NDVI and GDP per capita are changing in the opposite direction; if *r*_*NDVI*,*GDP*_>0, it shows that the change direction of NDVI and GDP per capita is the same. The closer *r*_*NDVI*,*GDP*_ is to 1, the more positive is the synchronism between GDP growth and NDVI change. t.

**Table 1:** Evaluation criteria for the types of impact of poverty alleviation policies on ecological health.

GDP per capita	NDVI	Development patterns under poverty alleviation policy
slope>0	slope>0	A
slope>0	slope<0	B
slope<0	slope>0	C
slope<0	slope<0	D

## Results

### Spatial distribution and change differences in the average annual NDVI in the fourteen contiguous areas of dire poverty

**Fig 3:**
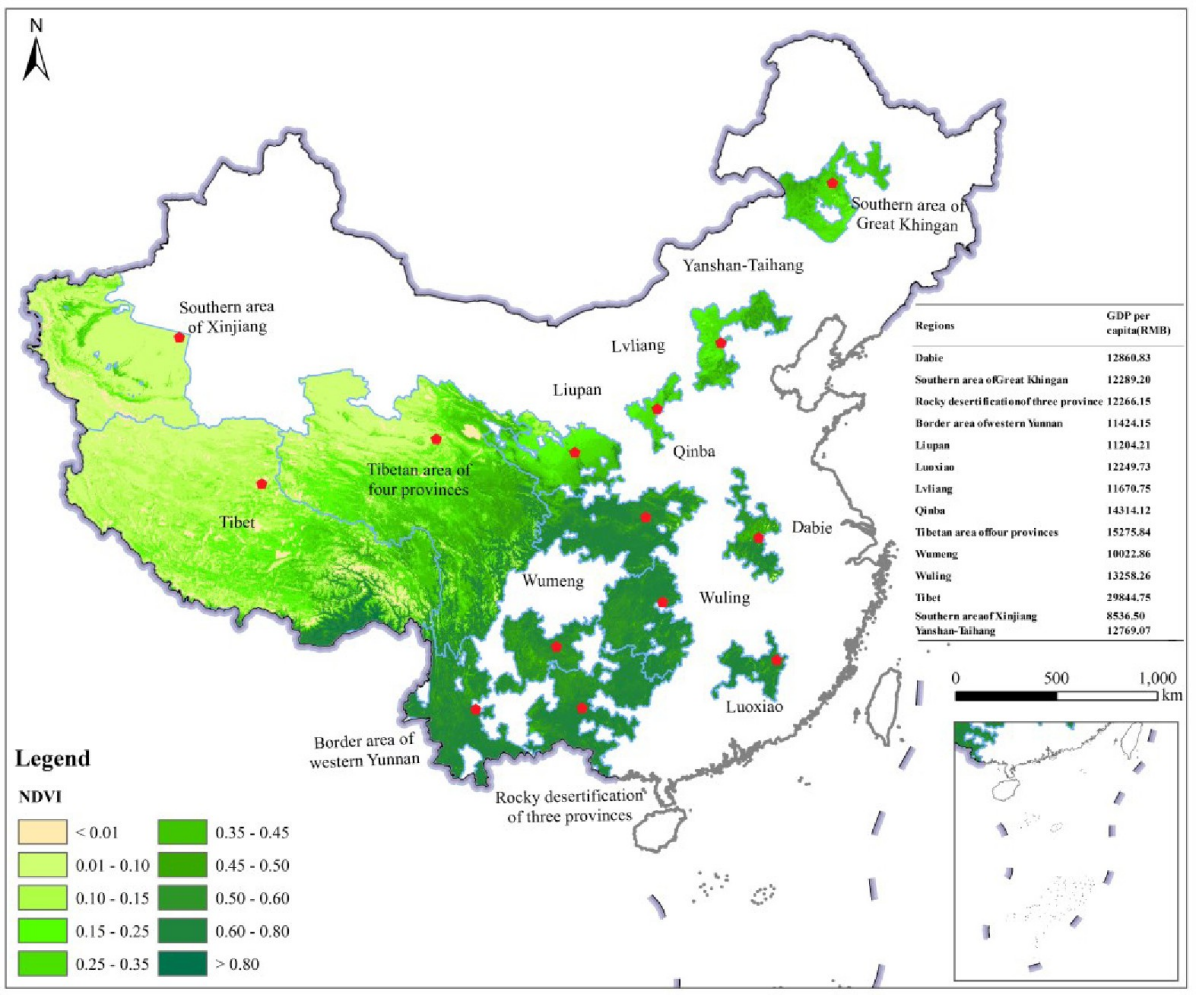
Spatial distribution of average annual NDVI and GDP per capita (RMB) in the fourteen contiguous areas of dire poverty between 2000 and 2019.

**Fig 4:**
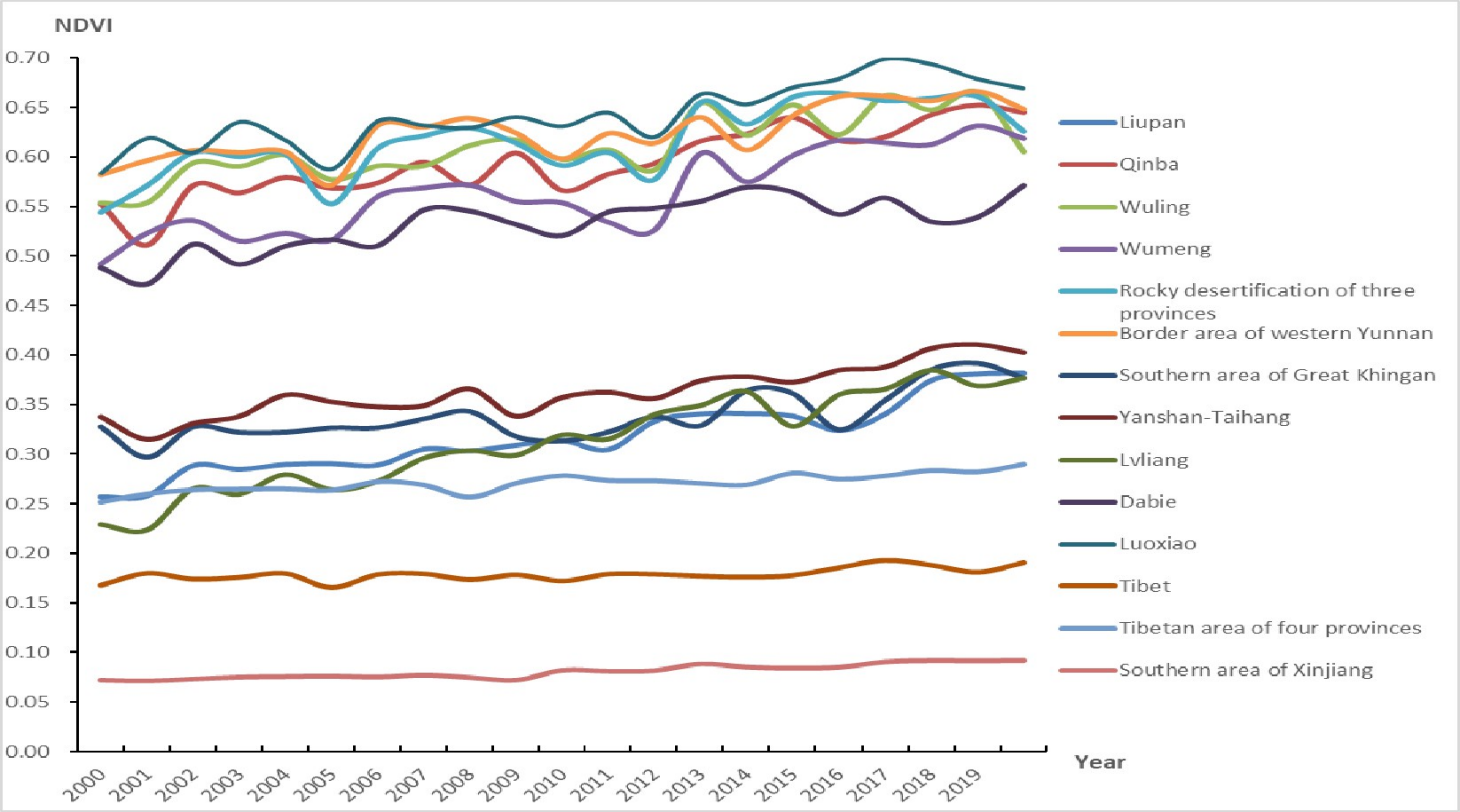
Average annual NDVI in the fourteen contiguous areas of dire poverty from 2000 to 2019.

The value range of the average annual NDVI in the fourteen contiguous areas of dire poverty was between -0.17 and 0.89 at the cell level between 2000 and 2019, which showed significant spatial heterogeneity and generally showed a decreasing trend from the contiguous poverty areas located in southern and eastern China to those located in western and northern China (Fig 3). The values of the average annual NDVI were larger than 0.50 in the Luoxiao, Border area of western Yunnan, in the Rocky desertification of three provinces, Wuling, Qinba, Wumeng and the Dabie areas; they ranged from 0.20 to 0.50 in the Yanshan-Taihang, the southern area of Great Khingan, Liupan, Lvliang and Tibetan area of four provinces; and were less than 0.20 in Tibet and the Southern area of Xinjiang between 2000 and 2019 (Figs 3 and 4).

The values of the average annual NDVI in the fourteen contiguous areas of dire poverty in China showed an overall increasing trend from 2000 to 2019 (Fig 4), with an increasing rate of 0.86% per year. Among them, the value of the average annual NDVI in the Lvliang Mountain area showed the greatest rate of incrase, accounting for 3.03% per year, and that in the Liupan Mountain area showed the second-highest rate of increase, accounting for 2.42% per year. The increased rates of average annual NDVI in the Yanshan-Taihang area, the southern area of Xinjiang, Wuling, Wumeng, and the Rocky desertification of the three provinces were between 1.00% and 2.00% per year, and those in other contiguous poverty areas were less than 1.00% per year from 2000 to 2019 (Table 2).

**Table 2:** The change differences and correlations between GDP and NDVI in the fourteen contiguous areas of dire poverty.

Poverty area	GDP per capita increase rate (%) per year	NDVI increase rate (%) per year
The whole area	43.81	0.86
Tibet	26.38	0.38
Southern area of Great Khingan	30.46	0.97
Luoxiao	36.78	0.81
Yanshan-Taihang	41.33	1.09
Tibetan area of four provinces	48.18	0.61
Southern area of Xinjiang	50.06	1.33
Dabie	52.47	0.52
Wuling	53.21	1.01
Border area of western Yunnan	55.83	0.73
Wumeng	56.86	1.42
Qinba	60.81	0.90
Liupan	63.78	2.42
Rocky desertification of three provinces	68.53	1.07
Lvliang	85.00	3.03

### Spatial distribution and change differences of average annual GDP in the fourteen contiguous areas of dire poverty

**Fig 5:**
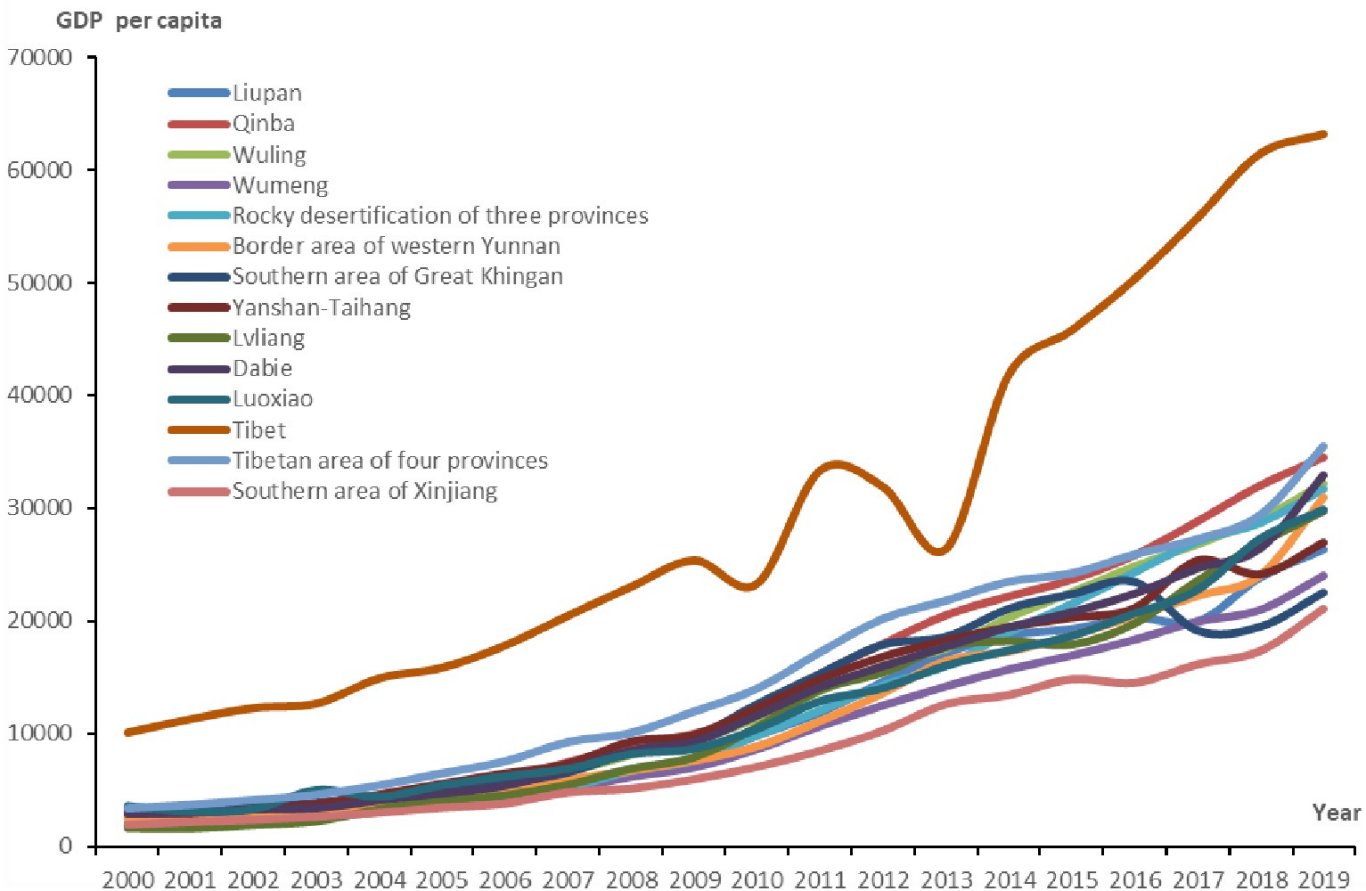
Changes in GDP per capita in the fourteen contiguous areas of dire poverty from 2000 to 2019.

The GDP per capita in the fourteen contiguous areas of dire poverty showed a rapid growth trend between 2000 and 2019, increasing from 3,030.08 RMB to 29,577.59 RMB, accounting for 43.81% per year (Fig 5). The annual GDP per capita of the Tibetan area showed the highest increase, increasing by 53,124.00 RMB, and that of Lvliang was the largest increase rate, increasing by 85.00% per year from 2000 to 2019.

The average annual GDP per capita in the fourteen contiguous areas of dire poverty was more than 20,000.00 RMB in 2019 (Fig 3), but in 2000, the GDP per capita of all these regions was less than 10,000.00 RMB, except in Tibet. Among them, the average annual GDP per capita increased to 63,192.50 RMB in the Tibet poverty area. The increased rate of average annual GDP per capita was greater than 50% per year in the 8 contiguous poverty areas of the rocky desertification of three provinces, Liupan, Qinba, Wumeng, area of western Yunnan, Wuling, Dabie and Southern area of Xinjiang. They increased by 68.53%, 63.76%, 60.81%, 56.86%, 55.83%, 53.21%, 52.47% and 50.06% per year, respectively, during the period from 2000 to 2019 (Table 2 and Fig 5).

### Response relationship between NDVI change and GDP growth in the fourteen contiguous areas of dire poverty

**Fig 6:**
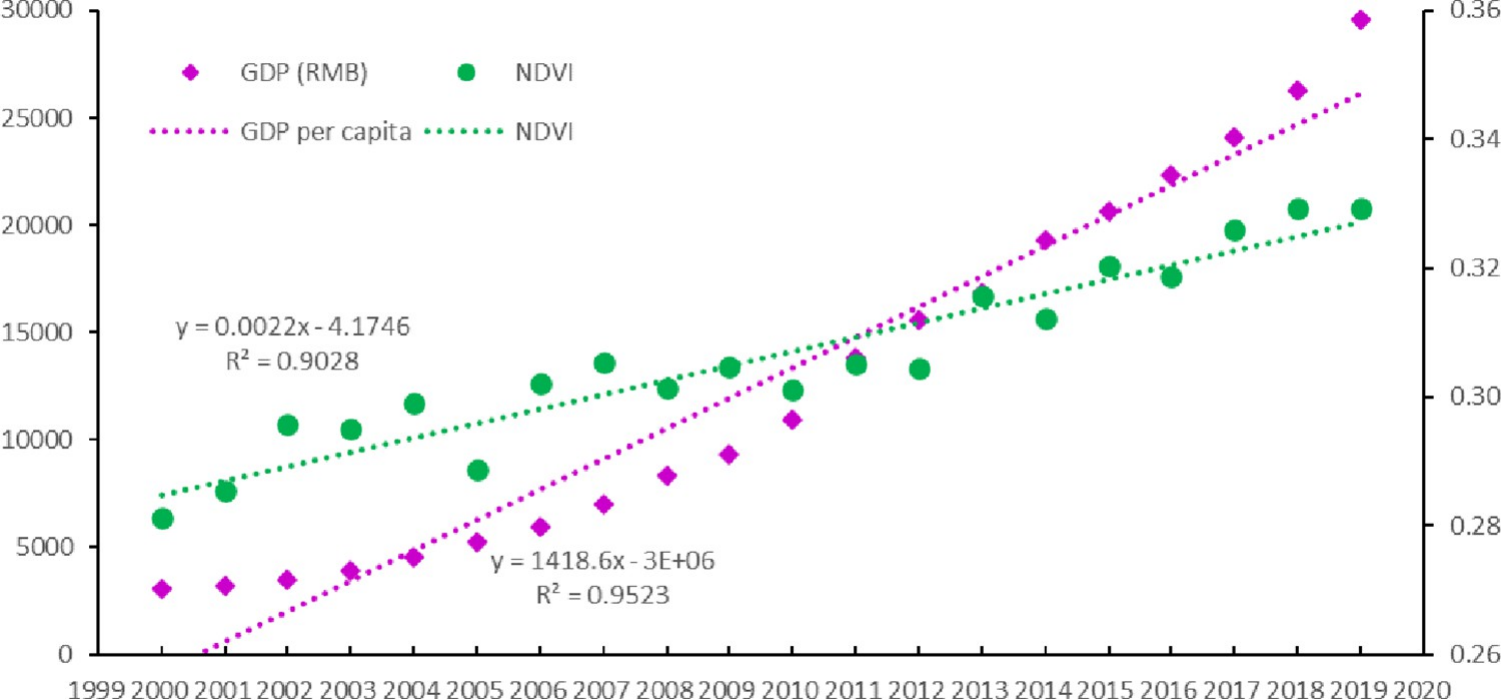
Change trends of NDVI and GDP in the fourteen contiguous areas of dire poverty from 2000 to 2019.

During the period from 2000 to 2019, the evaluation of the impact of poverty alleviation policies on ecological health in the whole poverty area showed that development took place according to a coordinated and sustainable (A) development pattern. The computed results of the response relationship of the average annual NDVI and GDP per capita in the whole poverty area showed that the correlation coefficients between the average annual GDP and NDVI with time (year) reached 0.95 and 0.90, respectively, which indicates that there was a significant increasing trend in both the average annual NDVI and GDP per capita during the period from 2000 to 2019 (Fig 6). Moreover, the results of the Pearson correlation coefficient between the average annual NDVI and GDP per capita in the fourteen contiguous areas of dire poverty reached 0.94 between 2000 and 2019, which indicated that the economic and ecological health of fourteen contiguous areas of dire poverty were developing in coordination. This meant that the poverty alleviation policy of the whole poor area was sustainable, which not only promoted economic development but also promoted the development of ecological health.

During the period from 2000 to 2019, the impact of poverty alleviation policies on ecological health in the whole poverty area was evaluated. The results showed that the development patterns of the fourteen contiguous areas of dire poverty were coordinated and sustainable. The Pearson correlation coefficients between GDP per capita and NDVI in the poverty areas of the southern areas of Xinjiang, Lvliang, Liupan and Yanshan-Taihang were 0.95 (p<0.01), 0.94 (p<0.01), 0.94 (p<0.01) and 0.90 (p<0.01), respectively, which meant that the development of economic and ecological health in these areas was synchronous. The Pearson correlation coefficients between GDP per capita and NDVI in the poverty areas of the southern area of Great Khingan, Tibet and Dabie were less than 0.7, which were 0.64 (p<0.01), 0.68 (p<0.0.1) and 0.69 (p<0.01), respectively, and those in the other contiguous poverty areas were between 0.70 and 0.90 (Table 2). This meant that the economic and ecological health development in these areas was less synchronized.

To better understand the impact of poverty alleviation policies on ecological health in the same region at different times, the period from 2000 to 2013 was regarded as one of normal poverty alleviation, and the period from 2014 to 2019 was regarded as one of targeted poverty alleviation. The impact of regional ecological health on regional economic development was compared and analyzed before and after the implementation of targeted poverty alleviation. According to Table 3, the development patterns of most contiguous areas of dire poverty were the coordinated and sustainable (A) development patterns during 2000–2013 and 2014–2019. Especially with the implementation of the targeted poverty alleviation policy of China relaunched in 2013, both NDVI and GDP per capita showed a significant increasing trend in the contiguous poverty areas of Wuling, Wumeng, Rocky desertification of three provinces, the Border area of western Yunnan, Yanshan-Taihang and the Tibet area, where the positive response between average annual NDVI and GDP per capita was higher than that during the period from 2000 to 2013. In these regions, the Pearson correlation coefficient between GDP per capita and NDVI is also higher than it was from 2000 to 2013. This means that rapid economic growth not only did not cause ecological degradation but also pushed ecological environment development toward health and sustainability in these poverty areas. The average annual GDP per capita has increased rapidly in the contiguous poverty areas of Liupan, Qinba, Lvliang, Luoxia, the Tibetan area of four provinces, and the southern area of Xinjiang, in which the average annual NDVI showed a slowly increasing trend. Although these regions exhibited coordinated and sustainable (A) development patterns during 2000–2013 and 2014–2019, the GDP per capita and NDVI trends are less synchronized. The development pattern of Dabie during 2014–2019 was one of economic growth (B), which means that its ecological environment and economic development were going in the opposite direction.

**Table 3:** Pearson correlation and development pattern between NDVI and GDP per capita in the fourteen contiguous areas of dire.

Region	Parameter	2000–2013	2014–2019	2000–2019
Whole area	NDVI slope	0.0019**	0.0029**	0.0022
	GDP slope	1083.62**	1979.49**	29577.59**
	Develop pattern	A	A	A
	Pearson correlation	0.79**	0.90*	0.94**
Liupan	NDVI slope	0.0053**	0.0068	0.0054**
	GDP slope	1100.61**	1377.58**	1338.63**
	Develop pattern	A	A	A
	Pearson correlation	0.90**	0.90*	0.94**
Qinba	NDVI slope	0.0044**	0.0046	0.0053**
	GDP slope	1338.60**	2391.40**	1747.81**
	Develop pattern	A	A	A
	Pearson correlation	0.67**	0.62	0.88**
Wuling	NDVI slope	0.0045**	0.0033	0.0049**
	GDP slope	1154.84**	2343.84**	1602.48**
	Develop pattern	A	A	A
	Pearson correlation	0.67**	0.68	0.85**
Wumeng	NDVI slope	0.0046*	0.0061*	0.0061**
	GDP slope	926.61**	1538.44**	1202.23**
	Develop pattern	A	A	A
	Pearson correlation	0.56*	0.87*	0.86**
Rocky desertification of three provinces	NDVI slope	0.0038*	0.0024	0.0050**
	GDP slope	1057.40**	2579.09**	1615.22**
	Develop pattern	A	A	A
	Pearson correlation	0.44	0.61	0.79**
Border area of western Yunnan	NDVI slope	0.0030*	0.0071	0.0036**
	GDP slope	973.98**	2164.63**	1379.56**
	Develop pattern	A	A	A
	Pearson correlation	0.53*	0.69	0.78**
Southern area of Great Khingan	NDVI slope	8.6770E-4	0.0080	0.0029**
	GDP slope	1250.14**	192.35	1241.75**
	Develop pattern	A	A	A
	Pearson correlation	0.25	-0.34	0.64**
Yanshan-Taihang	NDVI slope	0.0028**	0.0065**	0.0038**
	GDP slope	1218.74**	1445.75**	1369.42**
	Develop pattern	A	A	A
	Pearson correlation	0.71**	0.84*	0.90**
Lvliang	NDVI slope	0.0087**	0.0050	0.0078**
	GDP slope	1227.98**	2108.40**	1479.26**
	Develop pattern	A	A	A
	Pearson correlation	0.92**	0.68	0.94**
Dabie	NDVI slope	0.0054**	-0.0044	0.0036**
	GDP slope	1164.98**	2269.85**	1509.31**
	Develop pattern	A	B	A
	Pearson correlation	0.80**	-0.71	0.69**
Luoxiao	NDVI	0.0035**	0.0056	0.0048**
	GDP	960.32**	2338.46**	1348.30**
	Develop pattern	A	A	A
	Pearson correlation	0.66*	0.60	0.88**
Tibet	NDVI slope	3.1884E-4	0.0018	6.4017E-4**
	GDP slope	1712.05**	5707.20**	2800.52**
	Develop pattern	A	A	A
	Pearson correlation	0.35	0.57	0.68**
Tibetan area of four provinces	NDVI slope	0.0013**	0.0022*	0.0012**
	GDP slope	1432.29**	2003.24**	1665.49**
	Develop pattern	A	A	A
	Pearson correlation	0.66*	0.63	0.81**
Southern area of Xinjiang	NDVI slope	9.3224E-4**	9.9533E-4	0.0011**
	GDP slope	734.72**	1231.11**	978.53**
	Develop pattern	A	A	A
	Pearson correlation	0.89**	0.80	0.95**

## Discussion

### Method of evaluating the effect of poverty on ecological health

The evaluation of poverty alleviation policies has been a hot topic in recent decades. Many scholars focus on comprehensive assessments from the perspective of income growth, income gap, and the number of poor people [4,39]. However, few researchers have evaluated the impact of poverty alleviation policies from the perspective of ecological health. Based on GDP per capita and NDVI data, this paper establishes a method for evaluating the effect of poverty alleviation policies on ecological health. This method was used to evaluate the impact of poverty alleviation policies on ecological health in 14 poverty-stricken areas in China. The evaluation results are basically in line with the situation of the fourteen contiguous areas of dire poverty. However, this method is only a preliminary exploration of the impact assessment of poverty alleviation policies on ecological health, and there is still room for improvement. First, the evaluation indicators need to be improved. Due to the limitation of data availability, this paper uses GDP per capita and NDVI to represent economic development and ecological environment health, respectively. However, if possible, the changes in natural resources, environmental health and biodiversity can be used as indicators to judge the impact of poverty alleviation policies on ecological health. Second, the evaluation indicators need to be adjusted according to the different geographic conditions, economic baseline conditions and functional positioning of the different regions. For example, in some areas with poor environments, the restoration of the ecological environment is slow. At this time, the assessment of the impact of poverty alleviation policies on the ecological environment will produce errors. In some areas where the ecological environment is close to saturation, the ecological environment rises slowly. At this time, the assessment of the impact of poverty alleviation policies on the ecological environment will also produce errors. Third, due to the limitation of the data, this study cannot evaluate the degree of the impact of poverty alleviation policies on ecological health. Therefore, future research can select representative evaluation indicators for specific regions to evaluate the impact of poverty alleviation policies on ecological health.

### Impact of evaluation results on poverty alleviation policies

Contiguous poverty areas are mainly distributed in remote mountainous areas, hills, plateaus, high-risk areas of natural disasters, and minority population areas, where eco-environment vulnerability is normally higher than in other adjacent areas and is more sensitive to rapid economic growth [58,59]. Thus, these areas were selected to investigate the impact of poverty alleviation policies on ecological health. The analysis shows that the development patterns of these regions were coordinated and sustainable (A) from 2000 to 2019. Moreover, the consistency of economic and ecological health development during 2000–2013 was lower than during 2014–2019. Some research found that before 2013, the ecological environment quality and economic development in China’s poverty-stricken areas could not be synchronized, and the level of synchronization was poor [60,61]. Other scholars have found that after 2014, the economy and ecological health of some regions were highly coupled [62]. This was consistent with our research results and proved the effectiveness of the method for evaluating the effect of poverty alleviation policy on ecological health [63].

We set the coordinated and sustainable (A) development pattern as the best development pattern. If the development pattern of a region is evaluated as coordinated and sustainable (A), it means that the poverty alleviation policy of the region is sustainable, suitable for local development conditions and can be used continuously in the future. If the development pattern of a region is considered to be focused on economic growth (B), it means that the poverty alleviation policy of the region only takes into account economic development and ignores environmental protection and development. This is unsustainable and needs to be adjusted or modified in the future. If the development pattern of a region is evaluated as being an eco-environmental-dominant (C) pattern, it means that the poverty alleviation policies in the region have not lifted the people out of poverty. This is counterproductive to the goal of poverty alleviation and should be adjusted or modified in future policies. If the development pattern of a region is evaluated as one of morbid development (D), it means that the poverty alleviation policy in the region has not lifted the people out of poverty and has also destroyed the environment. This is the most unhealthy poverty alleviation measure, and the poverty alleviation policy needs to be revised immediately according to the natural and geographical conditions of the region. By using this method to evaluate the impact of poverty alleviation policies on ecological health, the effectiveness of poverty alleviation policies in poor regions of the world can be evaluated, which will help optimize poverty alleviation policies, eliminate poverty and promote sustainable development.

## Conclusions

The results of the analysis show that the development patterns of these regions were coordinated and sustainable (A) from 2000–2019 in all contiguous poverty areas of China. Moreover, the consistency of economic and ecological health development during 2000–2013 was lower than that during 2014–2019. However, the development pattern in Dabie during 2014–2019 was focused on economic growth (B). Therefore, it is necessary to adjust poverty alleviation policies according to coordinated and sustainable (A) development patterns to promote the highly consistent development of economic and ecological health. For the economic-growth (B), eco-environmental-dominant (C) and morbid (D) development patterns, it is necessary to change the poverty alleviation policies to promote regional sustainable development and achieve regional poverty alleviation.
